# The impact of neoadjuvant chemotherapy on the clinical efficacy and serum tumor marker levels in patients undergoing radical surgery for gastric cancer

**DOI:** 10.1097/MD.0000000000036040

**Published:** 2024-01-12

**Authors:** Bingqiang Li, Xuan Geng

**Affiliations:** a Department of Gastrointestinal Surgery, Xuzhou Central Hospital, Xuzhou, Jiangsu Province, China.

**Keywords:** carcinoembryonic antigen, clinical efficacy, Karnofsky Performance Status (KPS), neoadjuvant chemotherapy, radical surgery for gastric cancer, surgical indicators, tumor markers

## Abstract

The objective of this article is to study the impact of neoadjuvant chemotherapy (NAC) on the clinical efficacy and serum tumor marker levels in patients undergoing radical surgery for gastric cancer (GC). Thirty patients who underwent routine radical surgery for GC in our hospital from January 2020 to June 2021 were included in the control group. Thirty patients who underwent radical surgery for GC after receiving NAC from July 2021 to December 2022 were included in the observation group. The treatment outcomes of the observation group were assessed and analyzed. The surgical indicators, tumor markers, Karnofsky Performance Status (KPS), and occurrence of adverse reactions were compared between the 2 groups. Comparisons were made between the 2 groups in terms of surgical duration, number of lymph node dissections, intraoperative blood loss, time to postoperative ambulation, length of hospital stay, and time to postoperative passage of flatus (*P* > .05). The observation group had a higher proportion of R0 resection at the surgical margin compared to the control group (*P* < .05). The serum tumor markers of the 2 groups were compared before treatment (*P* > .05). After treatment, the levels of serum carcinoembryonic antigen, alpha-fetoprotein, cancer antigen 125, and carbohydrate antigen 72-4 decreased in both groups, and the observation group showed a greater reduction in these tumor marker levels compared to the control group (*P* < .05). The KPS scores of the 2 groups were compared before treatment (*P* > .05). After treatment, the KPS scores increased in both groups, with the observation group showing a higher improvement compared to the control group (*P* < .05). The overall incidence of adverse reactions, including incision infection, pleural effusion, pulmonary infection, intestinal obstruction, and gastric emptying disorders, was lower in the observation group (6.67%) compared to the control group (26.67%) (*P* < .05). The combination of NAC with radical surgery for GC is safe and feasible. It can significantly increase the R0 resection rate, effectively improve the levels of serum tumor markers, enhance patient’s quality of life, and result in fewer surgical adverse reactions.

## 1. Introduction

Gastric cancer (GC) is one of the leading causes of cancer-related deaths worldwide. Although the global incidence rates have been declining, there are still over 782,000 deaths annually.^[1]^ The incidence and prevalence of GC vary by region, and the incidence rate among males is twice as high as that among females. GC has the highest incidence rates in East Asia, Central Europe, and Eastern Europe, accounting for approximately 87% of global cases.^[2]^ Although surgery is currently the preferred treatment method for GC, the 5-year overall survival (OS) rate after surgery is only 20% to 30%, indicating poor prognosis overall. Therefore, the medical field has been actively exploring treatment methods to improve the prognosis of GC.^[3,4]^

Neoadjuvant therapy has been used for the treatment of various other cancers, such as non-small cell lung cancer and head and neck cancer.^[5,6]^ Furthermore, the feasibility of neoadjuvant chemotherapy (NAC) for GC is also being actively explored. The MAGIC trial is the first phase III randomized controlled trial that provided evidence of the superiority of NAC. Its results showed that NAC significantly improved disease-free survival and OS in GC.^[7]^ With the successive success of studies such as FLOT4,^[8]^ CROSS,^[9]^ RESOLVE,^[10]^ and PRODIGY,^[11]^ the importance of NAC as a treatment strategy for improving the prognosis of GC has been further confirmed. NAC has been incorporated into the GC treatment guidelines of many countries.^[12–14]^ However, there is still a lack of clinical data on NAC for treating GC in our country. Therefore, the aim of this study is to investigate the impact of NAC on the clinical efficacy and serum tumor marker levels in patients undergoing radical surgery for GC. The goal is to provide more data support for clinical treatment of GC.

## 2. Data and methods

### 2.1. General data

The study was approved by the Ethics Committee of Xuzhou Central Hospital. Thirty patients who underwent routine radical surgery for GC in our hospital from January 2020 to June 2021 were included in the control group. Thirty patients who received NAC and subsequently underwent radical surgery for GC from July 2021 to December 2022 were included in the observation group. In the control group, there were 17 male patients and 13 female patients. Their ages ranged from 39 to 72 years, with an average age of 58.69 ± 2.68 years. The tumor locations were as follows: 5 cases in the upper part of the stomach, 9 cases in the middle part of the stomach, and 16 cases in the lower part of the stomach. The tumor stages according to the International Union Against Cancer (UICC) classification were as follows: 16 cases of stage IIIB, 4 cases of stage IIIA, 5 cases of stage IIIB, and 5 cases of stage IIIC. The pathological classification included 22 cases of adenocarcinoma, 6 cases of signet ring cell carcinoma, and 2 cases of other types. In the observation group, there were 18 male patients and 12 female patients. Their ages ranged from 37 to 74 years, with an average age of 58.65 ± 2.71 years. The tumor locations were as follows: 6 cases in the upper part of the stomach, 9 cases in the middle part of the stomach, and 15 cases in the lower part of the stomach. The tumor stages according to the UICC classification were as follows: 16 cases of stage IIIB, 3 cases of stage IIIA, 6 cases of stage IIIB, and 5 cases of stage IIIC. The pathological classification included 23 cases of adenocarcinoma, 4 cases of signet ring cell carcinoma, and 3 cases of other types. A comparison was made between the 2 groups regarding general information such as tumor stage according to the UICC classification and tumor location (*P* > .05).

Inclusion criteria: (1) The patients were 35 to 75 years old. (2) The patient met the 2015 edition of the “Clinical Oncology Handbook”^[15]^ diagnostic criteria for GC, and was diagnosed as GC by gastroscopy, CT, and histopathology, UICC stage IIB to IIIC. (3) The patient had no history of chemotherapy before enrollment, and the liver and kidney could tolerate chemotherapy. (4) Patients without acute infection, no blood disease. (5) Patients with complete clinical data.

Exclusion criteria: (1) Pregnant and lactating women. (2) Patients with primary malignant tumors in other parts. (3) Coagulation dysfunction, hemorrhagic disease patients. (4) Patients with acute abdomen such as pyloric obstruction and gastrointestinal perforation.

### 2.2. Methods

#### 2.2.1. Neoadjuvant chemotherapy.

The NAC regimen used in this study was the S-1 and Oxaliplatin regimen. On the first day of chemotherapy, intravenous infusion of oxaliplatin injection (Aiheng, National Drug Approval Number H20040817) at a dose of 130 mg/m^2^ was administered. From day 1 to day 14 of chemotherapy, oral administration of S-1 Capsules (Aiyi, National Drug Approval Number H20100135) at a dose of 40 mg, twice daily, was given. Each treatment cycle consisted of 21 days, with a 7-day interval between 2 cycles. Radical surgery for GC was performed after 2 treatment cycles, preceded by a CT examination to assess the condition of the disease.

#### 2.2.2. Surgical methods.

Under general anesthesia, the patient is placed in a supine position. A midline incision of approximately 15 cm is made in the upper abdomen. For radical distal gastrectomy, the greater and lesser curvatures of the stomach are completely separated, and the corresponding blood vessels are dissected and ligated. After division of the duodenum and transection of the stomach, a standard Billroth I or Billroth II anastomosis is performed. For radical proximal gastrectomy, the greater and lesser curvatures of the stomach are completely separated, and the corresponding blood vessels are ligated, with clearance of the respective lymph nodes (LNs). Additionally, esophagogastrostomy is performed after transection of the esophagus and stomach. For total gastrectomy, during the dissection of the greater and lesser curvatures of the stomach, the corresponding blood vessels are ligated, with clearance of the respective LNs. After transection of the duodenum and esophagus, a Roux-en-Y anastomosis is performed between the esophagus and jejunum. After ligament division and LN clearance, a specimen retrieval and gastrointestinal reconstruction are performed through a midline incision of approximately 6 cm in the upper abdomen.

#### 2.2.3. Laboratory testing methods.

One week before surgery and 1 week after surgery, fasting blood samples are collected from the patients in the early morning. The samples are then centrifuged at 2000 rpm for 10 minutes at a low temperature. After centrifugation, the serum is collected and immediately stored at −80°C in a freezer for further testing. The serum levels of carcinoembryonic antigen (CEA), alpha-fetoprotein (AFP), cancer antigen 125 (CA125), and carbohydrate antigen 72-4 (CA72-4) are detected using the enzyme linked immunosorbent assay (ELISA) method. The procedure involves taking out serum samples and adding 50 μL of diluted standard samples and test samples into each well. Then, antibodies are added to the wells. After incubating at 37°C for 1 hour and subsequent washing, affinity-biotinylated lectin-horseradish peroxidase is added and the incubation continues at 37°C for 30 minutes. After another round of washing, substrate A and B are added, and the mixture is incubated at 37°C for 10 minutes. Finally, stop solution is added to terminate the reaction. The optical density 450 value is measured using a microplate reader (ELISA reader). The specific microplate reader mentioned, the SPECTRA MAX190, is manufactured by Molecular Devices, a company based in the United States. The ELISA kit used for the detection is purchased from Biolegend, and the experiment is conducted strictly following the instructions provided in the kit’s manual.

### 2.3. Observed indexes

(1) The treatment outcomes of the observation group were assessed. Complete remission: disappearance of all lesions and maintained for at least 4 weeks. Partial remission: reduction of the maximum diameter of lesions by at least 50% and maintained for at least 4 weeks. Stable disease: reduction of the maximum diameter of lesions by <50% or an increase of <25%. Disease progression: appearance of new lesions or an increase in the maximum diameter of lesions by at least 25%. The overall response rate was calculated as the sum of the complete remission rate and the partial remission rate. The disease control rate was calculated as the sum of the complete remission rate, partial remission rate, and stable disease rate. (2) A comparison was made between the 2 groups regarding intraoperative blood loss, surgical duration, surgical margin, number of LNs dissected, postoperative ambulation time, postoperative hospital stay, and postoperative time to passage of flatus. (3) The levels of serum CEA, AFP, CA125, and CA72-4 were compared between the 2 groups. (4) The Karnofsky Performance Status (KPS) scores were compared between the 2 groups. The KPS score ranges from 0 to 100, where 0 represents death and 100 represents normalcy without any symptoms. A higher score indicates better quality of life for the patients. (5) A comparison was made between the 2 groups regarding the occurrence of adverse reactions such as incision infection, pleural effusion, pulmonary infection, intestinal obstruction, and delayed gastric emptying.

### 2.4. Statistical processing

Data analysis was performed according to SPSS 24.0 software. The measurement data included age, surgical indicators, etc. The data were expressed as ($\bar{x}\pm s$), and the *t* test was used to analyze the difference of indicators. Count data include: treatment effect and gender, etc. χ^2^ test analysis of differences between groups of indicators.

## 3. Results

### 3.1. Effect of NAC in the observation group

Among the 30 patients in the observation group who received NAC, clinical symptoms improved to varying degrees. Specifically, 5 patients achieved complete remission, 14 patients achieved partial remission, 9 patients had stable disease, and 2 patients experienced disease progression. The overall response rate was 63.33% (19/30), and the disease control rate was 93.33% (28/30).

### 3.2. Comparison of surgical indicators between the 2 groups

The 2 groups were compared in terms of surgical duration, number of LNs dissected, intraoperative blood loss, postoperative ambulation time, postoperative hospital stay, and postoperative time to passage of flatus (*P* > .05). The observation group had a higher proportion of R0 resection compared to the control group (*P* < .05). See Table 1.

**Table 1:** Comparison of surgical indicators between the 2 groups ($\bar{x}\pm s$).

Group	Surgical duration (min)	Intraoperative blood loss (mL)	Surgical margin (%)		Number of lymph nodes dissected (piece)	Postoperative ambulation time (h)	Postoperative time to passage of flatus (h)	Postoperative hospital stay (d)
			R0 resection	R1 or R2 resection				
Observation group (n = 30)	235.44 ± 30.25	137.27 ± 42.69	25 (83.33)	5 (16.67)	29.52 ± 5.52	26.34 ± 7.55	61.24 ± 10.58	11.12 ± 7.25
Control group (n = 30)	225.54 ± 28.41	120.34 ± 38.52	18 (60.00)	12 (40.00)	30.48 ± 5.84	26.15 ± 7.46	61.50 ± 9.84	10.98 ± 7.33
*t*	1.307	1.613	4.022		−0.654	0.098	−0.099	0.074
*P*	.196	.112	.045		.515	.922	.922	.941

### 3.3. Comparison of serum tumor markers between the 2 groups before and after treatment

The serum tumor marker levels were compared between the 2 groups before treatment (*P* > .05). After treatment, the serum levels of CEA, AFP, CA125, and CA72-4 decreased in both groups. However, the observation group had a greater reduction in these tumor markers compared to the control group (*P* < .05). See Table 2.

**Table 2:** Comparison of serum tumor markers between the 2 groups before and after treatment ($\bar{x}\pm s$).

Group	n	CEA (ng/mL)		CA125 (U/mL)		AFP (μg/L)		CA72-4 (kU/L)	
		Before treatment	After treatment	Before treatment	After treatment	Before treatment	After treatment	Before treatment	After treatment
Observation group	30	37.10 ± 2.47	16.89 ± 2.36*	62.61 ± 5.44	32.54 ± 4.33*	4.48 ± 1.46	1.31 ± 1.22*	9.34 ± 2.75	4.36 ± 1.32*
Control group	30	37.22 ± 2.50	20.19 ± 2.57*	62.43 ± 5.37	37.85 ± 4.63*	4.39 ± 1.51	2.24 ± 1.31*	9.25 ± 2.83	5.42 ± 1.53*
*t*		−0.187	−5.180	0.129	−4.588	0.235	−2.846	0.125	−2.873
*P*		.852	.000	.898	.000	.815	.006	.901	.006

AFP = alpha-fetoprotein, CA125 = cancer antigen 125, CA72-4 = carbohydrate antigen 72-4, CEA = carcinoembryonic antigen.

*indicate statistically significant.

### 3.4. Comparison of KPS scores between the 2 groups before and after treatment

The KPS scores were compared between the 2 groups before treatment (*P* > .05). After treatment, the KPS scores increased in both groups, with the observation group showing higher scores compared to the control group (*P* < .05). See Table 3.

**Table 3:** Comparison of KPS scores between the 2 groups before and after treatment ($\bar{x}\pm s$).

Group	n	KPS score	
		Before treatment	After treatment
Observation group	30	65.87 ± 2.53	75.56 ± 3.21*
Control group	30	65.54 ± 2.45	73.48 ± 2.97*
*t*		0.513	2.605
*P*		.610	.012

KPS = Karnofsky Performance Status.

* Compared with the same group before treatment *P* < .05.

### 3.5. Comparison of adverse reactions between the 2 groups

The overall incidence of adverse reactions such as incision infection, pleural effusion, pulmonary infection, intestinal obstruction, and delayed gastric emptying was lower (6.67%) in the observation group compared to the control group (26.67%) (*P* < .05). See Table 4.

**Table 4:** Comparison of adverse reactions between the 2 groups [n (%)].

Group	n	Incision infection	Pulmonary infection	Pleural effusion	Delayed gastric emptying	Intestinal obstruction	Overall incidence
Observation group	30	0 (0.00)	0 (0.00)	1 (3.33)	0 (0.00)	1 (3.33)	2 (6.67)
Control group	30	1 (3.33)	2 (6.67)	2 (6.67)	1 (3.33)	2 (6.67)	8 (26.67)
*χ*^2^							4.320
*P*							.038

## 4. Discussion

In terms of incidence and mortality rates, GC remains a major global health problem. Radical surgical resection, combined with comprehensive multidisciplinary approaches, is crucial for achieving optimal treatment outcomes in resectable GC patients. Neoadjuvant therapy can increase the possibility of multimodal combination treatment, especially when serious complications related to surgical treatment arise, which may hinder the timely and effective administration of adjuvant therapy.^[15–16]^ Although there is currently a lack of global consensus on the necessity, timing, and specific regimens of neoadjuvant therapy, from a clinical perspective, it has been observed that neoadjuvant therapy can reduce the incidence of associated complications before performing extensive surgery on patients. It also provides an opportunity to assess the tumor’s biological characteristics. Additionally, neoadjuvant therapy contributes to downstaging of the tumor and increasing the rate of R0 resection.^[17]^ This study found that the clinical response rate after NAC was 63.33%, and the disease control rate was 93.33%. These results suggest that NAC can significantly reduce tumor volume and achieve tumor downstaging. By altering the biological characteristics of the tumor, NAC induces significant tumor cell death, leading to a reduction in tumor volume. The size of the tumor volume is a major determinant in tumor staging assessment. Therefore, NAC can effectively downstage the tumor, thereby increasing the likelihood of achieving R0 resection.

According to the GC treatment guidelines,^[18]^ commonly used observation indicators to evaluate the clinical efficacy of surgery include surgical duration, intraoperative blood loss, number of LN dissections, time for gastrointestinal function recovery, time for removal of drainage tubes, postoperative hospital stay, and postoperative complications (such as duodenal stump fistula, anastomotic leakage, postoperative bleeding, complications related to obstruction, traumatic pancreatitis, acute cholecystitis, incision-related complications, pulmonary-related complications, etc). These indicators are used to assess the surgical outcomes and clinical effectiveness of the procedure. In accordance with the guidelines, this study analyzed a selection of representative indicators and found no significant differences in surgical parameters between the 2 groups. However, there was a significant difference in postoperative adverse reactions, with a lower incidence observed in the NAC group. This suggests that NAC does not affect the surgical parameters directly associated with the procedure but may influence the occurrence of postoperative adverse reactions related to the surgery itself. It is known that factors such as tumor histological differentiation and grade, depth of invasion, LN metastasis, tumor node metastasis staging, and tumor length often play important roles in influencing the occurrence and severity of postoperative complications.^[19]^ Larger tumor volume is often associated with more advanced staging, which can lead to challenges in achieving complete resection. In addition, larger tumors may have characteristics such as infiltration into surrounding tissues and local LN metastasis, which further contribute to the complexity of the surgical procedure. These factors can increase the difficulty of achieving a curative surgery and subsequently lead to a higher incidence of postoperative complications. Preoperative NAC aims to achieve tumor downstaging by reducing tumor volume. It also induces significant tumor cell death, thereby disrupting its infiltration into surrounding tissues. This lays a solid foundation for the success of subsequent curative surgery and reduces the incidence of severe postoperative complications.

Serum tumor markers play an important role in the diagnosis, prognosis prediction, and recurrence monitoring of gastrointestinal malignancies.^[20]^ In addition, KPS score is a significant indicator for assessing the functional status of cancer patients and has important implications for predicting surgical outcomes.^[21]^ Research has shown that AFP is associated with the prognosis of GC patients who undergo surgery alone.^[22]^ Preoperative CEA can predict the prognosis of GC patients without LN metastasis.^[23]^ CA125 is associated with R0 resection rate, recurrence rate, peritoneal dissemination, and OS in unresectable advanced or recurrent GC patients.^[24,25]^ CA72-4 is related to pathology tumor node metastasis staging in GC patients.^[26]^ This study found that after treatment, the levels of serum CEA, AFP, CA125, and CA72-4 decreased significantly, and the KPS scores increased, with the observation group showing a greater magnitude of change. These findings suggest that curative surgery is effective in treating GC, improving the prognosis of patients and enhancing their quality of life. The combination of NAC and curative surgery has shown better outcomes in the treatment of GC, with improved postoperative recovery and prognosis. Although different serum tumor markers represent different meanings, they are closely related to the prognosis of cancer patients. Poor results of serum tumor markers may predict poorer pathology tumor node metastasis staging, lower R0 resection rate, higher postoperative recurrence rate, higher incidence of distant metastasis, and shorter survival time, indicating a poorer prognosis for cancer patients. In addition, the growth of tumors requires a large amount of energy substrates (such as glucose and fat), leading to cancer cachexia, which not only compromises the body’s functions but also hinders recovery, thereby reducing the quality of life and further worsening the prognosis of patients. Although radical surgery for GC removes the main tumor burden and improves patient prognosis, there may still be microscopically invisible residual lesions, leading to tumor recurrence or distant metastasis. Patients treated with NAC have a reduced likelihood of residual micro-metastases after surgery, thereby reducing the occurrence of tumor recurrence or distant metastasis. This is reflected in the significant decrease in serum tumor marker levels. By removing potential tumor burdens, the occurrence of tumor-related cachexia is also reduced. The improvement in KPS levels further supports this finding.

In summary, NAC combined with GC radical surgery is safe and feasible. It significantly improves the R0 resection rate, effectively improves the levels of serum tumor markers, and enhances the quality of life for patients while reducing surgical adverse reactions. However, our study has several limitations, such as the lack of comparison of the 5-year survival rates between the 2 groups. Further research based on this study will be conducted to address these limitations and delve deeper into the topic.

## Author contributions

**Conceptualization:** Xuan Geng.

**Data curation:** Bingqiang Li, Xuan Geng.

**Formal analysis:** Bingqiang Li.

**Investigation:** Xuan Geng.

**Methodology:** Bingqiang Li.

**Supervision:** Xuan Geng.

**Writing – original draft:** Bingqiang Li.

**Writing – review & editing:** Bingqiang Li, Xuan Geng.
